# The Involvement and Therapy Target of Immune Cells After Ischemic Stroke

**DOI:** 10.3389/fimmu.2019.02167

**Published:** 2019-09-11

**Authors:** Zhihong Jian, Rui Liu, Xiqun Zhu, Daniel Smerin, Yi Zhong, Lijuan Gu, Weirong Fang, Xiaoxing Xiong

**Affiliations:** ^1^Department of Neurosurgery, Renmin Hospital of Wuhan University, Wuhan, China; ^2^State Key Laboratory of Natural Medicines, School of Basic Medical Sciences and Clinical Pharmacy, China Pharmaceutical University, Nanjing, China; ^3^Department of Pharmacology and Toxicology, Shandong Institute for Food and Drug Control, Jinan, China; ^4^Department of Neurosurgery, University of Central Florida College of Medicine, Orlando, FL, United States; ^5^Central Laboratory, Renmin Hospital of Wuhan University, Wuhan, China

**Keywords:** ischemic stroke, immune cell, macrophage, microglia, inflammation

## Abstract

After ischemic stroke, the integrity of the blood-brain barrier is compromised. Peripheral immune cells, including neutrophils, T cells, B cells, dendritic cells, and macrophages, infiltrate into the ischemic brain tissue and play an important role in regulating the progression of ischemic brain injury. In this review, we will discuss the role of different immune cells after stroke in the secondary inflammatory reaction and focus on the phenotypes and functions of macrophages in ischemic stroke, as well as briefly introduce the anti-ischemic stroke therapy targeting macrophages.

## Introduction

Stroke is one of the major diseases threatening physical and mental health, with the characteristics of high morbidity, mortality, disability rate and recurrence rate, and brings heavy burden to families and society (1). Stroke is divided into two types, namely ischemic stroke (cerebral infarction) and hemorrhagic stroke (cerebral hemorrhage), of which 85% is ischemic stroke. Cerebral ischemia may result in secondary brain injury and neuronal death, producing inflammatory mediators and leading to inflammation in brain tissue (2).

Following cerebral ischemia-reperfusion, reactive oxygen species (ROS) cause initial breakdown of the blood brain barrier (BBB) by upregulating inflammatory mediators and activating matrix metalloproteinases (MMPs). The initial BBB breakdown occurs within 3 h after stroke onset and is accompanied by vasogenic edema. Destruction of the BBB induced by stroke promotes the migration of immune cells to the brain (3). Acute brain injury causes the release of damage associated molecular patterns (DAMPs) and the contents from dying and necrotic neurons into the extracellular environment, and subsequently trigger intense innate immune response involving microglia and infiltrating leukocytes (4). Ischemic stroke results in up-regulated expression of integrin on the leukocyte surface and of the corresponding adhesion molecules on the endothelium. Leukocytes wrap around the vascular endothelium before being activated by chemokines. Subsequently, the leukocytes firmly adhere to the endothelium and undergo either transcellular or paracellular diapedesis through the endothelial layer (5). In the early stages of cerebral ischemia, leukocytes are recruited by cell-adhesion molecules expressed on endothelial cells and enter parenchyma at a later stage. Recruitment of leukocytes, including neutrophils, monocytes and lymphocytes, is a continuous process and has a significant impact on the pathogenesis of ischemic brain injury (6).

Microglia in central nervous system (CNS) and peripheral immune cells, including blood-derived monocytes/macrophages, neutrophils, and lymphocytes, are recruited into the ischemic cerebral hemisphere which induce inflammatory response (4, 7). The inflammatory cascade in brain tissue could accelerate, expand or delay, alleviate ischemic brain injury (8). There are two main sources of macrophages infiltrating into ischemic brain tissue after stroke: microglia derived macrophages (MiDM) and monocytes derived macrophages (MoDM). Microglia originate from the migration and differentiation of macrophages during the primitive hematopoiesis of the fetal yolk sac, and they are resident into the brain in the early stage of fetal development, and maintain the proliferation ability during the postnatal development. By contrast, granulocyte-monocyte progenitors are the precursors of macrophages during development and adulthood (9). After ischemic stroke, peripheral monocytes are migrated through the BBB to the ischemic brain under the action of chemokines and cell adhesion molecules. Therefore, a series of changes of macrophages after stroke and their effects on disease progression are extremely complex.

In this review, we will discuss the role of different types of immune cells in the secondary inflammatory responses after stroke. We focus on the different phenotypes and functions of macrophages in ischemic stroke and briefly introduce the anti-cerebral ischemia therapy targeting macrophages.

## Immune Cells in Brain After Ischemic Stroke

**Figure 1:**
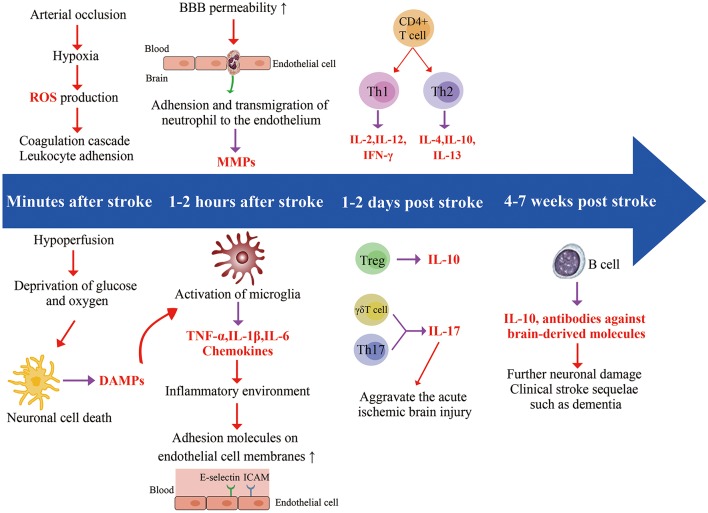
Timeline related developments regarding cell location and expression of different factors. Ischemic stroke starts in the blood vessels, where arterial occlusion results in hypoxia, reactive oxidative species (ROS) production, and coagulation cascade. In addition, ischemia also impacts the brain parenchyma. Hypoperfusion causes deprivation of glucose and oxygen, leading to a series of interconnected events (acidosis, oxidative stress, excitotoxicity, and inflammation), eventually causing neuronal cell death. The dying and dead neurons release danger-associated molecular patterns (DAMPs) which result in the activation of microglia. The release of chemokines and cytokines (TNF-α, IL-1β, IL-6) from microglia generates an inflammatory environment featuring activated leukocytes, and the increased expression of adhesion molecules on endothelial cell. Neutrophils enter the brain as early as 1 h after stroke and increase blood-brain barrier permeability by secreting matrix Metalloproteinases (MMPs), further aggravating ischemic injury. T cells have a damaging effect in this acute phase of stroke. Th17 cells and γδT cell further increase neutrophilic activity and aggravate the acute ischemic through the production of IL-17. B cells produce antibodies against brain-derived molecules, resulting in further neuronal damage in 4–7 weeks following stroke onset, possibly leading to clinical stroke sequelae such as dementia.

After acute stroke, multiple immune cells can enter the brain parenchyma in an orderly manner. Microglia increased in the early stage after stroke, and peripheral immune cells, including myeloid dendritic cells, monocytes/macrophages, and neutrophils, appeared within 1 day after stroke, peaked 3 days after stroke, and lasted until 7 days after stroke (10, 11). Subsequently, small increases of T and B lymphocytes were detected (Figure 1). Neutrophils are the first leukocytes subset to appear in the ischemic brain, which are detected within the first hour. These neutrophils remain in the cerebral microvessels, from where they damage the BBB by releasing ROS and proteolytic enzymes (12). Neutrophils penetrate the CNS parenchyma mainly following the more damaging second opening of the BBB, resulting in severe endothelial damage, destruction of adjacent blood vessels, and in some cases hemorrhagic transformation (13). Peripheral monocytes can be recruited to the ischemic brain within hours after BBB damaged. T lymphocytes influx into the brain within hours after tMCAO and aggregate around the border of the infarcted region. Particularly, cytotoxic CD8+ T cells have been indicated to be recruited into the brain as early as 3 h following stroke while CD4+ T cells are recruited within 24 h and peak at 72 h after reperfusion (14). There is increasing evidence that immune cells are involved not only in neuroinflammatory processes, but also in the maintenance of CNS homeostasis (Table 1).

**Table 1:** The different role of the cells involved in stroke pathology.

**Cells involved in stroke**		**Role in stroke pathology**
Neutrophils		Produce inflammatory factors, including MMP, iNOS, and ROS Aggravate BBB destruction Activate platelets and contribute to the thrombotic processes
T cells	CD4+ T cells	Mediate tissue repair through the production of IL-4 in synergy with macrophages
	CD8+ cytotoxic T cells	Lead to neuron death and aggravation of brain injury by cell interactions and release of perforin/granzyme after antigen-dependent activation
	Treg	Promote neuroprotection and repair
	γδT cells	Exacerbate ischemic damage through production of IL-17
	Th1 cells	Aggravate brain injury by secreting pro-inflammatory cytokines, including IL-2, IL-12, and IFN-γ
	Th2 cells	Exhibit neuroprotective effects on the injured brain by secreting anti-inflammatory cytokines such as IL-4, IL-5, IL-10, and IL-13
B cells		Attenuate inflammation after stroke by producing anti-inflammatory cytokines such as IL-10, TGF-β
Dendritic cells		Activate and enhance antigen presentation to T cells
Macrophage/Microglia		Release cytotoxic substances, induce inflammation, and lead to cell death (M1 phenotype) Clear cell debris by phagocytosis and release nutritional factors (M2 phenotype)
Astrocytes		Promote neurotoxicity through glial scar formation and pro-inflammatory cytokine release (e.g., IL-15) Provide neuroprotection via angiogenic and synaptogenic effects, neurotrophin production, and reducing excitotoxicity

### Neutrophil

**Figure 2:**
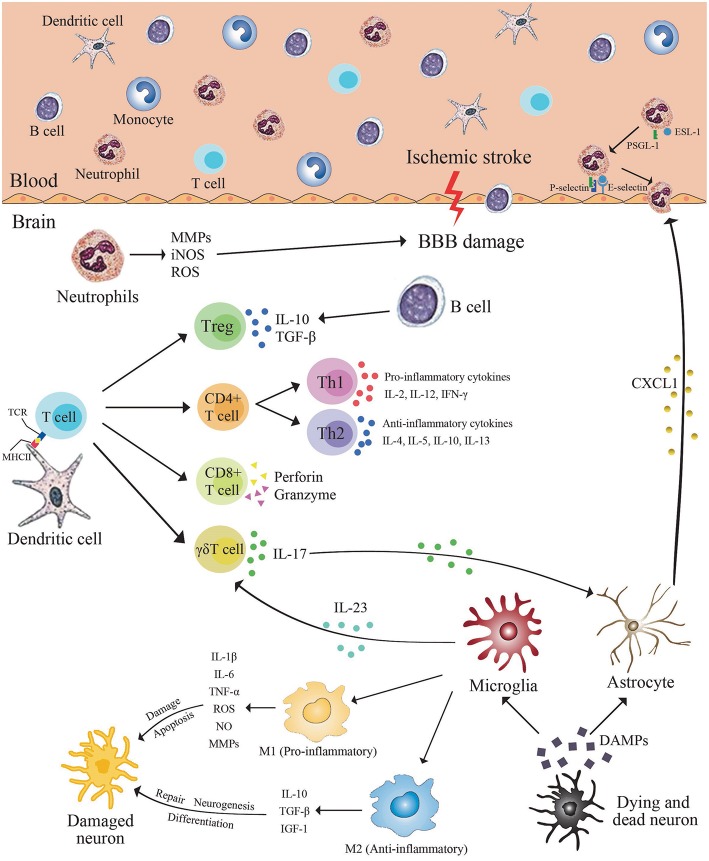
Immune cells in brain after ischemic stroke. Cerebral ischemic stroke leads to the release of danger-associated molecular patterns (DAMPs) from dying neurons. These molecules trigger the activation of resident microglia and astrocytes. Activated microglia release pro- or anti-inflammatory mediators resulting in neurons apoptosis or neurogenesis. In the ischemic brain, inflamed endothelial cells express adhesion molecules, such as intercellular adhesion molecule 1 (ICAM-1), P-selectin, and E-selectin, which recruit neutrophils and infiltration. Activated neutrophils infiltrating into the brain parenchyma produce a number of inflammatory factors, including matrix metalloproteinases (MMPs), inducible nitric oxide synthase (iNOS), and reactive oxygen species (ROS), which aggravate blood brain barrier destruction and cell death, as well as obstruct brain repair. Dendritic cells express major histocompatibility complex (MHC II) on the cell surface. Brain-derived antigens can be presented by MHC II on dendritic cell and can be recognized by receptors on the surface of T cells. Subsequently, the adaptive immune system is activated. Activated microglia/macrophages may stimulate activated CD4+T cells to differentiate into Th1 or Th2 cells, and then produce pro-inflammatory or anti-inflammatory cytokines to damage or protect the brain. CD8+ cytotoxic T cells lead to neuron death and aggravation of brain injury by cell interactions and release of perforin/granzyme after antigen-dependent activation. γδT cells caused damage to the surrounding tissues by secreting IL-17. B cells attenuate inflammation after stroke by producing anti-inflammatory cytokines such as IL-10 and TGF-β.

Neutrophils are one of the first cells to respond to cerebral ischemic injury, but their exact role in stroke pathology remains unclear. Although the phagocytosis of neutrophils may contribute to the removal of necrotic cell debris and promote tissue healing, its destructive effects lead to collateral tissue damage, BBB disruption, and brain edema (15). Infiltrating neutrophils have a significant destructive potential in many ways. Activated neutrophils infiltrating into the brain parenchyma produce a number of inflammatory factors, including matrix metalloproteinases, inducible nitric oxide synthase (iNOS) and ROS (16), which aggravate BBB destruction and cell death, as well as obstruct brain repair (Figure 2). In addition, the accumulation of neutrophils in blood vessels can damage local blood flow, leading to the absence of reflow in the affected microcirculation (17).

After stroke, necrotic lesions produce a great quantity of damage associated molecular patterns (DAMPs) (18), such as mitochondrial DNA (19), carboxyl alkylpyrrole (20), and ATP (21). Injured brain cells are exposed to inflammatory mediators, such as platelet activating factor (PAF), IL-1β, TNF-α, and chemokines (22). Subsequently, cell adhesion molecules (CAMs) were induced to express on the endothelial surface (23). The roll of neutrophils depends mainly on selectin, whereas adhesion and migration depend on integrin (24). Once the CAMs present on endothelial cell surface, they would interact with complementary receptors on the surface of the neutrophils. For example, P-selectin interact with its ligand P-selectin glycoprotein ligand 1 (PSGL1) resulting in neutrophils rolling on the endothelial surface along the flow direction (22), and the interactions between macrophage adhesion molecule 1 (MAC-1) of neutrophil and endothelial intercellular adhesion molecule 1 (ICAM-1) contributes to the adhesion of neutrophils to activated endothelium (25).

### T Cells

There is increasing evidence that immune cells are involved not only in neuroinflammatory processes, but also in the maintenance of CNS homeostasis. T cells are an important group of immune cells involved in the pathogenesis of certain neurological diseases by inducing innate or adaptive immune responses. After stroke, T cells preferentially migrate to the edge of the lesion, and the number of cells increases several days after ischemia, which can be detected in the brain parenchyma within 30 days after ischemia (26). Recombinant activated gene knockout (Rag^−/−^) mice lack the normal T cell and B cell function. Studies have shown that Rag^−/−^ mice has lower incidence of ischemic stroke. After Rag^−/−^ mice transferred with T cells, the infarction volume was increased significantly, indicating that T cells play an important role in the development of cerebral ischemic stroke (27, 28).

Approximately 40% of T cells infiltrating ischemic brain tissue are CD4+ helper T cells and about 30% are CD8+ cytotoxic T cells. Antibody mediated depletion of CD4+ or CD8+T cell subsets resulted in reduced infarct volume and secondary injury (29). When neuroinflammatory responses occur in the acute stage of cerebral ischemia, T cells act on neurons indirectly through the interaction with the innate immune system. After cerebral ischemia/reperfusion, activated and infiltrated microglia/macrophages may stimulate activated CD4+T cells to differentiate into Th1 or Th2 cells, and then produce pro-inflammatory or anti-inflammatory cytokines to damage or protect the brain (30). Th1 cells may aggravate brain injury by secreting pro-inflammatory cytokines, including IL-2, IL-12, and IFN-γ. Th2 cells may have neuroprotective effects on the injured brain by secreting anti-inflammatory cytokines such as IL-4, IL-5, IL-10, and IL-13 (31).

CD8+ cytotoxic T cells are the first T cell subsets to invade the ischemic brain and can be detected within a few hours after stroke (10). CD8+ cytotoxic T cells lead to neuron death and aggravation of brain injury by cell interactions and release of perforin/granzyme after antigen-dependent activation (29) (Figure 2).

Regulatory T cells (Treg) mainly express Foxp3 transcription factor, and inhibit excessive immune response by identifying autoantigens and foreign antigens. A large number of studies have shown that Foxp3+Treg have a protective effect in neuroinflammatory response after stroke (32). IL-10, an anti-inflammatory cytokine, is a key mediator of the neuroprotective function of Treg, which exerts anti-inflammatory effect by inhibiting IL-1β and TNF-α (33, 34). Studies have shown that intraventricular injection of IL-10 reversed the effects of Treg depletion (35). In addition, IL-10 mediates multiple biological functions and down-regulates more than 300 genes related to inflammatory pathways (36). However, it has also been found that Treg may affect BBB integrity in the acute stage of stroke, and may aggravate neuron injury by inducing microvascular dysfunction. The elimination of Treg within 24 h after middle cerebral artery occlusion (MCAO) reduced the amount of fibrin in mice and increased cerebral blood flow, indicating attenuated tissue injury.

γδT cells and Th17 cells caused damage to the surrounding tissues by secreting IL-17 which peaked on the third day after stroke (37). IL-17 specific antibody neutralization significantly improved the prognosis of stroke (38). In contrast to the adverse effects in the acute phase, IL-17 expression peaked for the second time around the 28th day after stroke, possibly due to the secretion of cytokines by reactive astrocytes to promote neurogenesis (37). This finding demonstrated that IL-17 promotes inflammatory response in the acute phase and promotes regenerative function in the later stage, indicating the complexity of adaptive immune response after stroke.

### B Cells

B cells have both protective and traumatic effects in cerebral ischemia. In mouse models, B cells are involved in chronic inflammation in the ischemic region where lymphoid tissue similar to B cell follicles is observed (39). B cells undergo isotype transformation, express plasma cell markers, secrete immunoglobulin, and affect long-term functional recovery after stroke. Consistently, oligoclonal immunoglobulin was found in the cerebrospinal fluid of some stroke patients, indicating that B cells in the CNS responded after stroke (40).

There is no clear consensus on the role of B cells in post-stroke recovery, although some animal studies have suggested a protective role of B cells in post-stroke injury. μMT^−/−^ mice were introduced a nonsense mutation into the transmembrane exon of IgM heavy chain, leading to B cell elimination (41). After middle cerebral artery occlusion, μMT^−/−^ mice exhibited larger infarct size, higher mortality, and more severe neurological deficits (42). Compared with wild type mice, the ischemic hemisphere of μMT^−/−^ mice showed more activated T cells, neutrophils, macrophages and microglia. Adoptive transfer of highly enriched populations of B cells from WT mice to μMT^−/−^ mice prior to stroke onset has protective effects that may be dependent on cytokine secretion. The same neuroprotective phenotype was not observed when adoptive transfer of B cells to IL-10 deficient mice (42). The above researches indicated that regulatory B cells provide neuroprotection through the IL-10 dependent mechanism.

Regulatory T cells and B cells attenuate inflammation after stroke by producing anti-inflammatory cytokines such as IL-10, transforming growth factor-β (TGF-β). In the pathological process of the CNS, IL-10 expressed in the brain increases regulatory lymphocytes and reduces the inflammatory response (39). Lipopolysaccharide (LPS) stimulation before adoptive transfer of B cells increases the production of IL-10, reduces infarct volume, increases the number of regulatory T cells, and inhibits the peripheral pro-inflammatory response (43).

Other animal studies applied Rag^−/−^ mice or mice only deficient in B cells, CD4+ T cells or CD8 T+ cells, to determine the contribution of specific lymphocyte populations to ischemia-reperfusion injury and recovery. Rag^−/−^ mice exhibited lower infarct volume and alleviated neurological deficits, but no improvement was observed in B-cell deficient mice after stroke, suggesting that B cells had no significant effect on the progression of infarction (27). In addition, B cell regeneration of Rag^−/−^ mouse had no significant effect on the neuroprotective effect after stroke (28).

Compared with other risk factors associated with cerebrovascular and degenerative dementia such as diabetes, hypertension, and hypercholesterolemia, the occurrence of stroke increases the susceptibility to dementia, especially vascular dementia (39, 44–46). However, the signaling pathways that lead to the progression of dementia after stroke are not fully understood. Recent studies have shown that cognitive impairment occurs 7 weeks after stroke in mice (39). Four to seven weeks after stroke, B cells congregate in the infarct area and produce IgA and IgG antibodies, which are associated with cognitive impairment. This has been demonstrated in four stroke models of two different mouse strains. Immunoglobulin synthesis was also found in cerebrospinal fluid of stroke patients for months after stroke (47, 48). These antibodies may lead to neuronal injury and cognitive impairment by binding to Fc receptors and activating the complement pathway (49). In addition, these antibodies worsen the disease by spreading into adjacent, unaffected healthy tissue.

### Dendritic Cells (DCs)

DCs are the most effective antigen presenting cells (APCs), which plays a crucial role between innate immunity and adaptive immunity (50). They act as an outpost of the immune system, including the CNS, constantly monitoring the environment. DCs are located near cerebrospinal fluid, but they may migrate to cervical lymph node, or trigger an immunogenic T cell response (51). Under physiological conditions, the presence of DCs in brain parenchyma is rare, but with the aggravation of neuroinflammatory reaction, DCs increases in brain parenchyma. Although DCs belong to a unique immune cell lineage with multiple phenotypes, it shares certain markers with macrophages, microglia, and monocytes. These cells express MHC II, and have similar morphological characteristics, which is not conducive to the accurate identification of cells (52).

After the experimental animal cerebral ischemic, it was found that cells with DC characteristics were present in their brains. Kostulas et al. reported the existence of MHC II (OX6+)-expressing cells in ischemic brain tissues of rats after permanent middle cerebral artery occlusion (pMCAO) (53). MHC II is expressed by DCs (OX62+) which are usually not present in the brain parenchyma but in the meninges and choroid plexus. DCs invaded the ischemic core during the first few hours after ischemia and gradually increased until 6 days after ischemia. They observed that DCs (OX62+) in brain parenchyma also expressed CD11b (OX42+), suggesting that some microglia developed into DCs after ischemic stroke (53). Reichmann et al. used photochemically induced cerebral ischemia in mice to detect the expression of DCs and demonstrated that DCs were existed in the periphery area of cerebral infarction and in degenerated cortical thalamic fiber bundles, as well as subcortical nuclei within a few weeks after stroke (54).

Recent studies have shown that, in addition to playing a role in the classical antigen-dependent immune response, DCs are able to take shape of immune responses locally without relying on migration to lymphatic organs or antigen presentation. Toll-like receptors interact with DCs through pathogen-associated molecular patterns (PAMPs) or DAMPs, leading to the rapid release of cytokines that initiate a innate immune cascade. For example, conventional DCs (cDCs) and monocyte-derived DCs (MoDCs) can release IL-23 under the stimulation of TLR5 ligands.

### Macrophages

Circulating monocytes migrate into the brain parenchyma via endothelial cells and differentiate into tissue macrophages. There are also a large number of macrophage-like cells in the CNS, namely microglia, which can also transform into activated macrophages during tissue damage (55). Many studies have found that microglia and monocytes/macrophages aggravate the inflammation and injury of stroke. Macrophages are the main cells of the immune system, playing an important role in the repair and regeneration of the CNS (56). In addition to the neuroprotective effect, macrophages are also the main source of pro-inflammatory cytokines, which can significantly inhibit brain tissue repair and neurogenesis (57). Thus, the dual functions of microglia and infiltrating macrophages hinders restoration after stroke.

Inflammatory microenvironment has a great influence on microglial/macrophage phenotype, leading to different gene expressions and biological functions in brain tissue. Studies have found that LPS induced microglia/macrophage M1 phenotype mainly releases cytotoxic substances, induces inflammation, and leads to cell death, which is manifested as destructive effects on the brain. Whereas, IL-4-induced M2 phenotype clears cell debris by phagocytosis and releases nutritional factors, which is manifested as neuroprotective effects on the brain (58). As a consequence, these results suggest that facilitating the M2 phenotype and suppressing the M1 phenotype are beneficial to brain recovery after stroke.

In addition, the promotion of effective neurogenesis has gradually become an important treatment method for stroke, because the dead neurons in the cortical lesion need to be replaced by new neurons to supplement and reconstruct the neuronal connections. Recent studies have shown that macrophages are involved in each step of neurogenesis. For instance, macrophages produce neurotrophic factors to induce cell migration (59), but they can also produce inflammatory cytokines that reduce the survival rate of newborn neurons (60). Therefore, understanding the roles and mechanisms of macrophages in neurogenesis after stroke will be helpful to find new therapies to promote brain repair.

## The Sources and Function of Macrophages After Ischemic Stroke

Macrophages infiltrating into ischemic brain tissue after stroke mainly include Microglia Derived Macrophages (MiDM) and Monocyte Derived Macrophages (MoDM). Activated MiDM and MoDM exhibit some of the same and different characteristics in cerebral ischemic injury. Common characteristics refer to the expression of the same phenotypic markers, the capability of polarizing to pro-inflammatory/ anti-inflammatory (M1/M2) phenotype, and phagocytic function, as well as the high degree of morphological plasticity. However, MiDM and MoDM are different in some aspects. First, MiDM derive from the fetal yolk sac, residence in the brain at the early stage of embryonic development, and maintain the ability of proliferation. In contrast, MoDM are derived from granulocyte monocyte progenitors during development and maturation (61, 62). After ischemic stroke, monocytes in peripheral blood migrate through BBB and are recruited into ischemic brain tissue under the action of chemokines and cell adhesion molecules. In addition, activation of MiDM is dependent on ATP/ADP signal, while MoDM can maintain viability and retain their functions in the absence of oxygen /ATP (62, 63). Finally, only MiDM have ramified morphology, of which the branches generate from the cell body and interact with surrounding neurons and other glial cells (64, 65). Although there are increasing evidences that MiDM are functionally different with MoDM, whether these differences signify that they play different roles in the progression and repair of brain injury remains to be confirmed (66, 67).

### Monocytes Derived Macrophages (MoDM)

**Figure 3:**
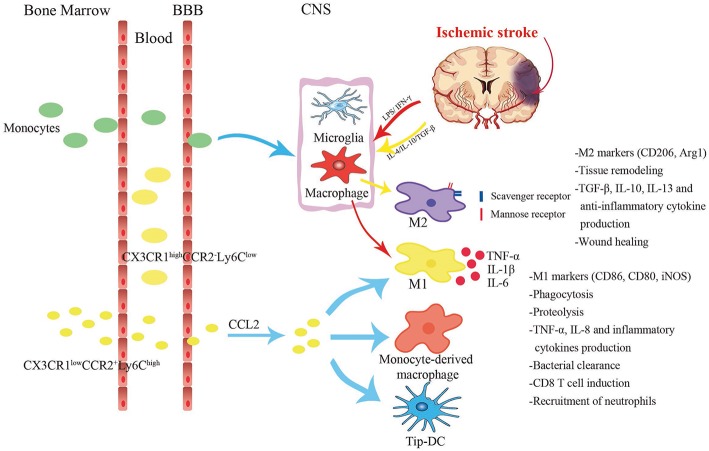
Different cell subsets changes in response to stroke. Two main subgroups of monocytes exist in the circulation, namely pro-inflammatory Ly6C^hi^CCR2^+^CX3CR1^lo^ monocytes and anti-inflammatory Ly6C^lo^CCR2^−^CX3CR1^hi^ monocytes. Ly6C^hi^CCR2^+^CX3CR1^lo^ monocytes infiltrate into the central nervous system from the blood via the CCL2-CCR2 axis, and differentiate into classically M1-like macrophages or Tip-DCs with strong phagocytosis. Under acute inflammatory circumstances, they turn into the direct precursors of macrophages in the peripheral blood. Anti-inflammatory monocytes are larger and act primarily as vascular patrols and induce neutrophil aggregation. After cerebral ischemia, the injury tissue releases various inflammatory cytokines. Lipopolysaccharide (LPS) and interferon-γ (IFN-γ) stimulate monocyte-derived macrophages to polarize toward M1 phenotype which secretes TNF-α, IL-1β, and IL-6. Alternative M2 is promoted by IL-4, IL-10, and TGF-β. It expresses substantial mannose receptors and scavenger receptors.

Monocytes are immune effector cells with surface pathogen recognition receptors and chemokine receptors that mediate the migration of monocytes from blood to brain after ischemic stroke. Under normal conditions, monocytes circulate in the bone marrow, blood, and spleen without proliferation. After acute cerebral ischemia, monocytes migrate from *in situ* to the ischemic area and accumulate in the ischemic region, then differentiate into macrophages (Figure 3). MoDM produce inflammatory cytokines, toxic molecules and are actively in phagocytosis (68). In the case of inflammatory response or pathogen infection, inflammatory factors, or pathogens bind to surface receptors of monocytes and mediate migration of monocytes to the injured tissues and differentiation into macrophages or inflammatory DCs (8). Monocytes are derived from macrophage-DC precursors (MDPs) and are constantly replenished by self-renewing hematopoietic stem cells (HSCs). Only in the case of injury can MoDM localize to the injured region and play an anti-inflammatory role by regulating the local activation of MiDM, thus becoming the driving force for the end of the immune response (69).

Different from MiDM, MoDM can transform metabolism into anaerobic mode in hypoxia/ischemia conditions, thus maintaining viability (62, 70). Many pathological processes, such as tumors, atherosclerosis, and cerebral ischemia, have hypoxic environment accompanied by the presence of macrophages (70). In recent years, studies have attempted to elucidate how macrophages adapt to hypoxic environment and some studies have found that hypoxia-inducible factors (HIF-1α) and transcription factor NF-κB are master regulators of this adaption (71, 72). The inflammatory response mediated by myeloid cells requires the participation of HIF-1α, and involves the reduction of iNOS expression and the decrease of ATP produced by glycolysis (73, 74). Hypoxia activates NF-κB and promotes the production of inflammatory factors (72). Hypoxia-induced expression of CXCL12 can regulate the mobilization and homing of HSC and progenitor cells to ischemic tissue (75, 76). Inflammatory MoDM express CCR2, CD11b, Ly6C, and low level CX3CR1 (77).

MoDMs are an important integral in tissue remodeling not only during the developmental stage but also in adulthood. In addition, macrophages inhibit inflammatory responses and autoimmune responses to autoantigens (78). MoDMs also control angiogenesis through various mechanisms, not only controlling vascular branches of the circulatory system, but also affecting lymphangiogenesis during development (79). Several recent studies have labeled blood monocytes with green fluorescent protein (GFP) to distinguish the roles of the two types of macrophages (80). The results showed that the infiltration of MoDM into ischemic brain reached its peak at 2–3 days after stroke (81–84). Some researchers demonstrated that in the rat models of transient MCAO (tMCAO) and pMCAO, monocytes began to infiltrate into the ischemic brain at 24–48 h after cerebral ischemia, but the number was lower than activated microglia (85, 86).

Fractalkine (CX3CL1) is a membrane binding chemokine, which is expressed by neurons in the normal CNS, while its receptor CX3CR1 is highly expressed on microglia. The interaction between CX3CL1 and CX3CR1 is essential to maintain the normal function of microglia in both physiological and pathological conditions (87). CCR2 is the receptor of monocyte chemotactic protein (MCP), which is expressed on many different types of cells, but primarily on the surface of monocyte. Different phenotypic monocyte subsets are distinguished according to the differences in CCR2 and CX3CR1 expression (Table 2). The monocyte subset in peripheral blood is distinguished by CX3CR1, which is phenotypically identified as LFA-1^+^, L-Sel^−^, Ly6C^−^, CCR2^−^, CX3CR1^hi^. While CCR2 marked “inflammatory” monocytes are LFA-1^−^, L-Sel^+^, Ly6C^+^, CCR2^+^, and CX3CR1^lo^ (88–90). In rodents, monocytes are divided into two main subsets according to chemokine receptor and Ly6C expression levels, namely pro-inflammatory subsets (Ly6C^hi^CCR2^+^CX3CR1^lo^) and anti-inflammatory subsets (Ly6C^lo^CCR2^−^CX3CR1^hi^; Figure 3). Analogously, human monocytes are highly homologous with murine monocytes and are divided into three subsets: the classical type (CD14^++^CD16^−^), the intermediate type (CD14^++^CD16^+^) and the non-classical type (CD14^+^CD16^++^). In terms of function, the mouse Ly6C^hi^ monocyte subset is similar to the human CD14^++^CD16^−^ and CD14^++^CD16^+^ monocyte subsets, and the Ly6C^lo^ monocyte subset is analogous to the human CD14^+^CD16^++^ monocyte subset (91).

**Table 2:** Characteristics and effects of distinct macrophages.

**Source**		**Phenotype**	**Identification markers**	**Functions**
Microglia		Resting state	Iba-1/ CD45^int^CD11b^+^F4/80	Determined by differentiation pattern (M1 or M2)
Macrophage/Microgila		Classical activation (M1)	CD16/CD32/CD86/CD80/iNOS/CD80	Pro-inflammatory
		Alternative activation (M2)	CD206/Arg1/Ym1/CD68	Anti-inflammatory
Monocyte	Mice	Pro-inflammatory monocyte	LFA-1^−^/L-Sel^+^/Ly6C^+^/CCR2^+^/CX3CR1^lo^	Clear dead cells Mediate inflammation
		Anti-inflammatory monocyte	LFA-1^+^/L-Sel^−^/Ly6C^−^/CCR2^−^/CX3CR1^hi^	Vascular surveillance
	Human	CD14^++^CD16^−^	CD93/CD64/CD11b/CD36 /CD62L CXCR1/CXCR2/CCR2^hi^	Similar to pro-inflammatory monocyte (mice)
		CD14^++^CD16^+^	CD74/CD40/CD54 CX3CR1^hi^/CCR2^hi^	Similar to anti-inflammatory monocyte (mice)
		CD14^+^CD16^++^	CX3CR1^hi^/CCR2^lo^	Similar to anti-inflammatory monocyte (mice)

CCR2 is associated with the recruitment, activation and tissue invasion of monocytes (92). Pro-inflammatory monocytes (Ly6C^hi^CCR2^+^CX3CR1^lo^) simultaneously express a large amount of CX3CR1 and CCR2, so that to have high mobility and can rapidly reach the inflammatory site during infection or tissue damage. In addition, it has been reported that Ly6C^hi^CCR2^+^ monocytes are direct precursors of microglia in blood circulation (83). Wattananit et al. found that blocking monocytes recruitment by the anti CCR2 antibody during the first week post ischemic stroke abolished long-term behavioral recovery, and drastically decreased the expression of anti-inflammatory genes (56). Chu et al. injected selective CCR2 receptor antagonist 1 h before MCAO, 2 and 6 h after MCAO, respectively, to prevent recruitment of Ly6C^hi^ monocytes to the brain. At 24 h after MCAO, worse functional outcome and extensive lesion was found (93). These results suggested that recruited pro-inflammatory monocytes have protective effects. In addition, studies have shown that the ablation of infiltrating monocytes leads to a decrease in cell with anti-inflammatory activity without affecting the expression of pro-inflammatory characteristic activities (56).

Anti-inflammatory monocytes (Ly6C^lo^CCR2^−^CX3CR1^hi^) express higher levels of CX3CR1 with longer half-life. They slowly invade the vascular endothelium and “patrol” the vascular system with blood flow in a way dependent on LFA-1 integrin and CX3CR1. The aim of this behavior is to remove damaged endothelial cells and thus maintaining the integrity of the vasculature (94). Recently, it has been reported that Ly6C^lo^ monocytes do not affect the progression and recovery of ischemic stroke (82). Furthermore, CX3CR1 also regulates the neurotoxicity of microglia in a cell-autonomous manner in neurodegenerative CNS diseases. The increase of soluble CX3CL1 enhances the inflammatory signals of microglia and macrophages expressing CX3CR1, leading to increased release of toxic cytokines and oxidative metabolites (95). In stroke models, inhibition of CX3CR1 signal transduction can reduce infarct area and improve neurological function, partly due to reduced neuroinflammatory responses (87). Tang et al. (96) and Denes et al. (97) found that the loss of CX3CR1 signal can reduce the proliferation of microglial cells/macrophages in the ischemic brain of mice, reduce the inflammatory response, and the peripheral recruitment of mononuclear macrophage subpopulation differentiation is also changed. Activation via TLR4 regulates the expression of CX3CL1 and CX3CR1 (98). Since the activation of CX3CR1 also promotes the NF-κB-dependent transcription, the signal transduction via CX3CR1 can promote the inflammatory effects induced by TLR4 activation, while the lack of CX3CR1 signal transduction can produce the opposite effect (87). Fumagalli S has indicated that the protective effects of CX3CR1^−/−^ mice in the early stage after ischemic injury and in the inflammatory response are related to a protective inflammatory environment, characterized by the promotion of the M2 polarization markers expression (99).

### Microglia Derived Macrophage (MiDM)

MiDMs are resident phagocytic cells in the brain, which contribute to tissue homeostasis by producing growth factors and clearing apoptotic cells. MiDMs have a large number of surface pathogen recognition receptors, which can conduct efficient phagocytosis and induce the production of inflammatory factors (100). *In vivo* lineage tracing studies showed that microglia originate from the primitive myeloid progenitor cells of the extraembryonic yolk sac that appeared before postnatal day 8 (P8), and entered the CNS after the angiogenesis of embryo on P9 (101, 102). Chemokine receptor CX3CR1 is structurally expressed in MiDM and can be induced in specific subsets of macrophages (100). Therefore, the high expression of CX3CR1 is considered as a selective marker of MiDM in the early stage of injury, and its main function in the brain is to support the communication between microglia and neurons. In addition, CD11b, F4/80, and low level CD45 were also expressed in adult microglia (77).

The severity of brain injury affects the proliferation of MiDM in the injured brain tissue. MiDMs exhibited significant proliferation after 30 min of transient ischemia, while no significant proliferation after 60 min of severe ischemia, resulting in a large area of microglia loss when supplementation with fresh microglia was restricted (85). MiDMs expend energy in an ATP-dependent manner to perform their normal functions, including production of inflammatory mediators and cytoskeletal recombination (103, 104). Therefore, after acute injury, MiDMs are easily affected by energy deficiency and alteration in local blood perfusion, affecting their reactivity and survival.

MiDMs and MoDMs are both powerful regulators of CNS repair/regeneration (64). However, both of these two cells appear to be double-edged swords in neurological restoration. MiDMs (GFP-negative) were rapidly activated at the first day after stroke, and a large number of MiDMs were observed until 4–7 days after ischemic stroke (105). After activation, MiDMs/MoDMs restore homeostasis and lighten the detrimental effects of inflammation by scavenging cell debris, eliminating local inflammation, and secreting nutrient factors that affect myelin formation and regulate cerebral angiogenesis (57). Activation of MiDMs also impede CNS repair and amplifies tissue damage (106). The existence of MiDMs relies on the colony factor 1 receptor (CSF1R) signal (89), the absence of which leads to the structural defects of the mice brain (107). MiDMs have been certified to promote neuronal viability (80), regulate neuronal excitability (108), and express a series of neuronal growth and survival factors, such as nerve growth factor (NGF) (80).

### Macrophages Phenotypes and Dynamic Changes After Ischemic Stroke

Once the brain environment is affected by pathophysiological changes such as stroke, MoDMs, and MiDMs can differentiate into macrophages which have special functions, including the production of inflammatory mediators and phagocytosis. The phenotypes of macrophages polarization are affected by immune stimulation, pathogenicity, and degree of injury. In general, activated MoDMs and MiDMs mainly differentiate into two phenotypes, the classical pro-inflammatory phenotype (M1) and the alternative anti-inflammatory phenotype (M2) (Figure 3). More precisely, classical M1 activation of macrophages stimulated by LPS and IFN-γ stimulation promotes the secretion of pro-inflammatory cytokines such as TNF-α, IL-1β, and IL-6 (109). High concentrations of proinflammatory cytokines in turn lead to plaque rupture and occlusive thrombosis. Studies have shown that complement C1q complex, which can induce polarization of macrophages toward pro-inflammatory M1-like phenotype, mediates synaptic pruning by tagging synapses and eliminating synapses by microglia phagocytosis (110). Furthermore, low levels of TNF-α, a classic M1 inflammatory cytokine, are known to promote synaptic connectivity under physiological conditions (111). All these studies indicate that M1 microglia have an adaptive role in regulating synaptic homeostasis and plasticity.

The M2 phenotype, induced by stimulation of IL-4, IL-10, TGF-β, express a large number of mannitol receptors and scavenger receptors, and secrete protective cytokines, thereby inhibiting the inflammatory response and promoting angiogenesis, as well as tissue reconstruction (112). Phenotypic markers of M1 include CD16, CD32, CD86, MHC II, and iNOS, while CD206, Arg1, and Ym1 are identified as M2 phenotype markers (113). However, recent studies have shown that the M1/M2 dichotomy is an oversimplified concept that represents only two extreme forms of activation. The status of MoDMs/MiDMs *in vivo* is much more complex than that *in vitro*, and may involve a series of phenotypes with different phenotypes but overlapping functions. For example, a large amount of evidences show that there is diversity in the M2 subgroup, such as M2a, M2b, M2c, and M2d, each of which has its unique physiological characteristics and biological function (114). The M2a subgroup was induced by IL-4/IL-13 and expressed mannose-CD206 receptor, showing strong anti-inflammatory properties and poor phagocytosis. The polarization of M2a is related to parasite immunity, Th2 cell recruitment, tissue repair, and growth stimulation. M2b cells exhibits both pro- and anti-inflammatory characteristics and is associated with immune memory response (114). M2c is induced by TGF-β/IL-10 and is mainly related to the clearance of cell debris, tissue healing, and the expression of Arg1, CD163, and CD206 (114, 115). The M2d subgroup highly expressed IL-10 and vascular endothelial growth factor (VEGF), and has a potential role in tissue repair. Different from other M2 macrophage subgroups, the M2d subgroup does not express Ym1, Fizz1, or CD206. Nevertheless, the broad classification of M1 and M2 still contributes to our understanding of the functional status of MoDMs/MiDMs during the progression of ischemic stroke.

It is worth noting that M1-polarized microglia/macrophages may play an essential role in the early repair process, which should not be overlooked. In contrast, a prolonged M2-dominated state after the acute pro-inflammatory period may lead to fibrosis and abnormal repair (116). Since both M1 and M2 phenotypes may play dual roles after brain injury, it is crucial to determine the time course of M1/M2 polarization after cerebral ischemia, so as to optimally switch microglia phenotypes appropriately and maximize the effects of tissue repair.

Under normal conditions, microglia are different from macrophages in morphology (117). The relative expression level of CD45 has been used to distinguish between resident microglia and newly recruited macrophages, both of which present CD11b^+^, whereas CD45 was highly expressed in macrophages (CD45^hi^) and moderately expressed in microglia (CD45^int^) (118). Consequently, microglia and monocytes/macrophages can be distinguished by CD11b^+^/CD45^int^ and CD11b^+^/CD45^hi^.

In different periods after cerebral ischemia, the polarization of macrophages toward different directions of M1/M2 is also different. Studies have shown that the number of CD206^+^/Iba-1^+^ M2-activated microglia peak 5 days after brain injury. Later, the CD16^+^/Iba-1^+^ M1-activated state dominates (119). Studies in a variety of CNS injury models showed that most newly recruited microglia/macrophages at the injury site expressed M2 gene, while microglia/macrophages expressing M1 gene dominated about 1 week after injury (120). This phenotypic conversion from M2-dominat to M1-dominat may result from a M2 to M1 shift within activated MiDMs/MoDMs, as well as the incessant recruitment of pro-inflammatory M1 MiDMs/MoDMs to the injured site. Initially, aged MiDMs/MoDMs were thought to be primed toward M1-polarization, dictating profound cytotoxicity and contributing to age-related deficits (121). Nevertheless, this notion was recently challenged by evidence of age-related microglia mainly primed toward the M2 phenotype (97), with downregulation of neurotoxic pathways and up-regulation of neuroprotective pathways. Further studies on the effects of aging on MiDMs/MoDMs polarization are critical to establishing therapies for age-related diseases such as stroke.

From day 3 after cerebral ischemia, the level of M1 gene gradually increased over time, and maintained an upward trend for at least 14 days after cerebral ischemia. In comparison, all the examined M2 markers began to express at 1–3 days after MCAO and peaked at 3–5 days following injury. Most M2-type genes started to decrease at 7 days after MCAO and were restored to pre-injury levels by day 14 (113, 120).

## Ischemic Stroke Therapy Targeting Macrophages

With the success of clinical trials of thrombolytic therapy and the extension of the therapeutic window of mechanical thrombolytic therapy recently, ischemic stroke treatment has made a breakthrough, controlling acute stage injuries and reducing mortality and long-term disability rates (122–124). There is increasing evidence indicates that MiDMs and MoDMs play an important role not only in neuroinflammation but also in subsequent tissue repair (125, 126). MiDMs and MoDMs, in addition to their role in phagocytosis of cell debris caused by brain injury, they also produce a variety of neurotrophic factors, angiogenic, and immunomodulatory factors which are involved in the regulation of brain remodeling and repair (127). Therefore, macrophages may be an effective target for the treatment of ischemic stroke.

Studies have shown that the neuroprotection and repair functions of macrophages depend on the recruitment and appropriate differentiation of inflammatory monocytes, which is of great significance for the exploration of new therapeutic targets. Until recently, inflammatory monocytes in the ischemic brain were considered to be the primary mediators of inflammatory CNS injury and secondary neurotoxicity. Therefore, it has been reported that therapeutic intervention through CCR2 at the level of inflammatory cell recruitment after ischemic stroke is beneficial (128, 129). It is worth noting that the above inflammatory CCR2+ monocytes with repair and protection effects do have temporary harmful effects, but the delayed protective functions that essential for secondary bleeding prevention and the neuroprotective function far outweigh its detrimental effects. Therefore, from a therapeutic perspective, completely blocking recruitment of early CCR2-dependent monocytes may not be appropriate, as it may also interfere with homeostasis and its protective function. Instead, the aim of therapeutic attempts should be to enhance the protective and repair effects of monocytes and macrophages in the inflammatory environment.

A large number of *in vivo* and clinical studies have shown the importance of inflammatory pathways in the pathogenesis of ischemic stroke (130). However, anti-inflammatory drugs such as glucocorticoid, COX2 inhibitors, and non-steroidal anti-inflammatory drugs have no significant effect (131), and increase the risk of cerebral blood vessels such as stroke, hemorrhage and atrial fibrillation (132, 133). However, non-selective cyclooxygenase inhibitors, such as aspirin, exhibit secondary prophylaxis, acting as anti-inflammatory agents by modulating NF-κB related genes (134). After stroke, the ischemic core develops into an irreversible inflammatory response lesion. The tissue around the ischemic core (penumbra) is also at risk of ischemia and inflammation. Therefore, saving penumbra and converting it to normal tissue is a key to the clinical treatment of stroke. The focus of most anti-inflammatory therapies is to protect the penumbra by preventing or slowing down the inflammatory cascade.

After stroke, immune cells, including monocytes/macrophages, infiltrate into the brain. Microglia and macrophages derived from monocytes in the brain are key players in the immune response for being activated and migrating to the injured tissue after stroke (135). Microglia are rapidly activated after brain injury and undergo morphological, functional, and behavioral changes, including the production of proinflammatory proteins, migration, proliferation, and phagocytosis (135). miR-124 is highly expressed in the nervous system and is considered to have anti-inflammatory effects (136). In the early stage of ischemic stroke, the damaged neurons released miR-124, inducing the polarization of myeloid cells from the initial state to the anti-inflammatory M2 phenotype (137). miR-155 is specifically expressed in hematopoietic cells and cells involved in vascular remodeling, including T cells, endothelial cells, and myeloid cells (138). It has been reported that tail vein injection of a specific miR-155 inhibitor 48 h after stroke in mice can promote functional recovery (139, 140). Inhibition of miR-155 may also attenuate the JAK/STAT inflammatory signal and M1 macrophage polarization by up-regulating SOCS1 and SHP-1 (138). Therefore, inhibition of miR-155 may suppress early reactive inflammation by enhancing BBB function and inhibiting M1 polarization, while at later stage, inhibition of miR-155 will be beneficial to control inflammation, remove necrotic debris, and have a repair effect (138).

The failure of stroke anti-inflammatory strategies may be related to the dual effects of microglia (141), therefore targeting the activation of microglia after stroke may be an effective way to reduce the brain injury caused by stroke. Minocycline treatment initiated 1 day after focal cerebral ischemia and lasted for 13 days decreased microglia activation, improved behavioral test scores, and increased survival rate (142). PARPs catalyze ADP ribose unit from NAD+ to target protein which include histones and transcription factors. Some studies have detected the applications of PARP inhibitors (e.g., olaparib and veliparib) after stroke, which found that they inhibit the activation of microglia and improve the survival rate of neurons (143, 144). Studies have shown that the application of histone deacetylase (HDAC) inhibitors—including trichostatin A and sodium butyrate—suppressed microglia activation and suppresses inflammatory markers in the ischemic brain, which also supports the beneficial effect of targeting inflammatory response (145). Mammalian target of rapamycin (mTOR) is a known immune response regulation factor. Inhibition of mTORC1 pathway by everolimus reduced microglia polarizing to pro-inflammatory M1 phenotype, alleviated secondary injury and improved motor functions, which directly acts of microglia and macrophages (146, 147) (Table 3).

**Table 3:** Ischemic stroke therapy targeting macrophages.

**Target**	**Mechanism of action**	**Therapeutic(s)**	**Effect(s)**
Inflammatory cascade	Non-selective cyclooxygenase inhibitors	Aspirin	Anti-inflammation by modulating NF-κB related genes Secondary prophylaxis
RNA therapy	miR-155 inhibitor	–	Attenuate the JAK/STAT inflammatory signal and M1 macrophage polarization by up-regulating SOCS1 and SHP-1
Microglia activation	Microglia inhibitor	Minocycline	Decrease microglia activation Improve behavioral test scores
	PARP inhibitors	Olaparib Veliparib	Inhibit the activation of microglia Improve the survival rate of neurons
	HDAC inhibitors	Trichostatin A Sodium butyrate	Inhibit microglia activation Suppress inflammatory markers
	mTORC1 inhibitor	Everolimus	Reduce microglia polarizing to M1 phenotype Alleviate secondary injury

## Expectation

As far as the current status of stroke treatment, only intravenous thrombolysis with tissue plasminogen activator (tPA) and endovascular thrombectomy are effective therapies to treat the acute phase. These approaches are limited by a narrow therapeutic window (<4.5 h for tPA and 6–24 h for endovascular treatment) (123, 124). Currently, there is no other effective method to treat cerebral ischemic stroke, so it is important to find new therapeutic strategies for cerebral ischemia.

In response to diverse microenvironmental cues, macrophages are polarized into different subsets and exhibit different functions. With a growing amount of studies authenticating the different contributions of MiDMs and MoDMs to CNS recovery, the recognition of extracellular signals triggering macrophage phenotypic transformation and the intracellular molecular switches could be promising therapeutic approaches for ischemic stroke. Understanding the dynamic changes of macrophage and time-dependent role of inflammatory mediators expressed by macrophage could help in searching new diagnostic, therapeutic, and prognostic strategies for post-stroke inflammatory reaction.

## Author Contributions

ZJ and RL wrote the initial draft. Figures and submission were prepared by XZ and YZ. DS and LG prepared the final version. XX and WF recommended a structure for the review, substantially advanced the draft.

### Conflict of Interest Statement

The authors declare that the research was conducted in the absence of any commercial or financial relationships that could be construed as a potential conflict of interest.
